# Geospatial analysis of flood emergency evacuation sites in Accra

**DOI:** 10.4102/jamba.v14i1.1172

**Published:** 2022-12-09

**Authors:** Rapheal Ane Atanga, Vitus Tankpa

**Affiliations:** 1 Department of Geography Education, Faculty of Social Sciences Education, University of Education, Winneba, Ghana; 2 Department of Tourism, School of Tourism and Hospitality, College of Business and Economics, University of Johannesburg, Johannesburg, South Africa; 3 Department of Environmental Science, Faculty of Environment, Harbin Institute of Technology, Harbin, China

**Keywords:** flood emergency evacuation centres, geographic information system, suitability sites, Accra Metropolitan Assembly, weighted linear combination method, remote sensing

## Abstract

Cities face frequent flood disasters with devastating impact on lives and property. Geospatial analysis using high elevation points can help to map out emergency evacuation centres for decision making on flood risk management. This research maps and analyses flood emergency evacuation centres in Accra. The objectives of this research were to map out flood hazard and vulnerable settlements, analyse the spatial distribution and identify the optimal locations for flood emergency evacuation sites. The data for this study comprises digital elevation models, stream networks, settlement data, flood risk zone maps of the study site and existing documents on flood disaster risk of the study area. This study adopted the weighted linear combination method to estimate site suitability for emergency evacuation sites. ArcGIS 10.6 was used to analyse the data. The research found that the optimal locations for potential evacuation centres are unevenly distributed between 300 m and 400 m above sea level, most which are in the north-eastern and north-western parts of Accra. The results can contribute to research, policy and decision-making on flood risk management in Accra and other localities with similar conditions. The research further contributes to literature on flood risk management and geographic information system.

**Contribution:**

This research is useful as mapping could increase awareness of evacuation sites to speed up rapid response to immediate impacts of flood events. The findings of the study will facilitate future decisions for demarcating potential flood emergency evacuation sites. The overall aim of this study is to contribute to literature, policy, spatial analysis and the practice of flood risk management in the study area. The research findings can also be applied to other flood-prone areas with similar contexts as the study site.

## Background and problem statement

Flooding constitutes one-third of natural disasters globally, and the risk of future floods is increasing because of climate and societal developmental changes (Jha, Bloch & Lamond 2012). Flooding in developing countries, especially in big cities, is a problem with increasing negative social, economic and environmental impacts (Adelekan 2010; Diagne 2007). The impacts of floods on developed and developing world nations vary in type and magnitude (Douglas 2017, Tschakert et al. 2010). Whereas flood impacts on advanced nations are mostly economic, the impacts on developing nations are high on humans, causing loss of life and ill health (Jha et al. 2012). Geographic information systems (GISs) play a crucial role in mapping, modelling and spatial analysis of flood disaster risk worldwide (Chang 2019; Ntajala et al. 2017; Opolot 2013).

The city of Accra in Ghana faces chronic floods with high recurrent impacts, thus impeding socio-economic development (Aboagye 2012). Studies on floods in Accra have focused on the causes of floods related to poor urban planning, encroachment on waterways and poor land use (Amoako & Boamah 2015; Karley 2009; Rain et al. 2011), but none have addressed the issue of mapping and spatial analysis of emergency evacuation sites for emergency response to flood disasters. Studies on spatial mapping and analysis of flood disaster risk on the study site are limited and did not include emergency evacuation sites for quick response. Nyarko (2000) mapped out flood risk zones in Accra using GIS. Again, Nyarko (2002) did a similar study in the same area and concluded that over 40% of Accra lies in high to medium flood risk zones. Jankowska, Weeks and Engstrom (2011) investigated vulnerability in relation to flooding, environmental degradation, social status, demographics and health in the slums of Accra using GIS and remote sensing techniques. Konadu and Fosu (2009) further applied the GIS model to predict the future extent of flood risk in Accra. Owusu et al. (2013) limited their study to a triangulated irregular network (TIN) and GIS analysis of reservoirs for flood management in Accra. There are also studies regarding geomorphological processes and GIS in relation to climate change’s impact on the study site (Gyekye 2013).

Apparently, no studies captured the element of emergency evacuation sites in relation to the flood-prone neighbourhoods in Accra. A GIS mapping and spatial analysis of emergency evacuation sites is useful for emergency flood response planning in Accra. Scholars argue that GIS mapping and analysis about distribution of emergency evacuation sites is critical in planning for rapid response to flood impacts on humans and movable property (Kourgialas & Karatzas 2011; Mustaffa et al. 2015). Geographic information systems are a useful tool for disaster risk management, and they are important for mapping and spatial analysis in recent years (Appeaning-Addo & Adeyemi 2013; Chang 2014, 2019). Hence, this study maps and analyses the distribution of potential evacuation sites within the Accra Metropolitan Assembly in the Greater Accra Region (GAR) of Ghana. This research is useful as mapping could increase awareness of evacuation sites to speed up rapid response to flood impacts during flood events. This can also facilitate future decisions for demarcating potential flood emergency evacuation sites. The aim of this study is to contribute to literature, policy, spatial analysis and the practice of flood risk management in the study area. The contribution can also be applied to other flood-prone areas with similar contexts as the study site.

### Research objectives

The specific objectives of this research are to:

- map out flood hazard and vulnerable settlement in the Accra Metropolitan Assembly
- analyse the spatial distribution and identify the optimal locations for flood emergency evacuation sites of flood-prone neighbourhoods in the Accra Metropolitan Assembly.

### Study site

**FIGURE 1:**
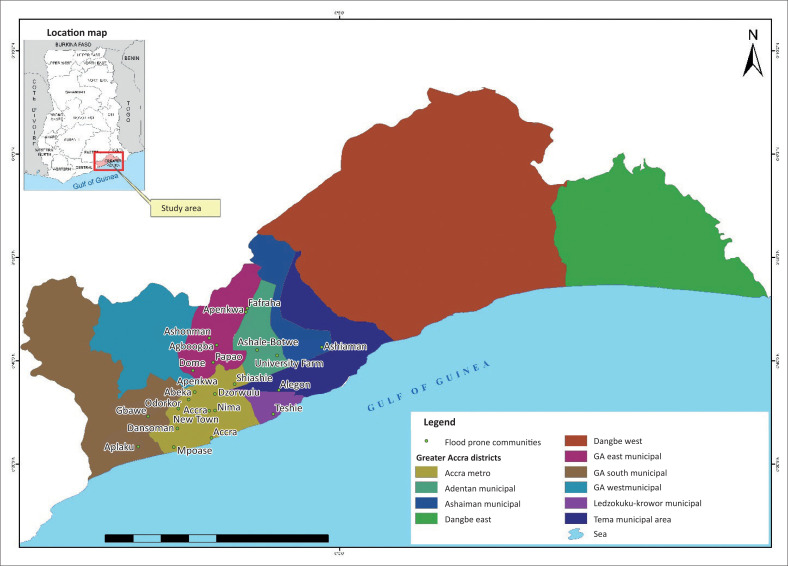
Study area.

The Accra Metropolitan Assembly in the GAR of Ghana was chosen for this research because of its chronic annual flood disasters in recent years. Rain et al. (2011) provided extensive mapping and analysis of uncontrolled urbanisation and flood risk in Accra and concluded that several pockets of the city are characterised by floods. Accra in the Metropolitan Assembly (AMA) is the capital of the GAR and the capital city of Ghana. It is the political administration of Ghana and the hub of international organisations in the country. In fact, Accra is the powerhouse of government activities and the city that links Ghana to overseas locations. The city has a population of about 2 million people with 11 sub-metropolitan assemblies under the AMA. Located along the Gulf Guinea Coast, the northern part of Accra is bordered by the Akwampim ranges with Tema Municipal Assembly bordering the eastern part. The topography of Accra is undulating, with lowlands dissected by seven drainage basins, namely Chemu, Osu, Songo Mokwe, Lafa, Sakumo, Densu, Kpeshie and Korle basins. The Korle basin experiences high run-off, resulting in frequent flood disasters in the city of AMA. Accra in the Metropolitan Assembly lies between −40 m below and 400 m above sea level. Thus, settlements along the drainage basins in low-lying areas experience frequent floods. These settlements are located within very high to medium flood risk zones, and they can be described as flood-prone communities. Such settlements experience floods annually. Figure 1 indicates Accra, labelled yellow in the legend within the GAR. The communities that are prone to flooding are named in Figure 1. Studies have established that these communities have had flood events leading to disasters (Rain et al. 2011). It is further evident that in Nyarko (2000, 2002), these places fall within drainage basins with high flood risk.

## Materials and methods

### Data sources and description

Data for this research were obtained from existing sources. The data for this study consisted of digital elevation models (DEMs), stream networks, settlement data, shapefiles of Ghana, flood risk zone maps of the study site and existing documents on flood disaster risk of the study area. Existing documents include literature such as Nyarko (2000, 2002) and Rain et al. (2011), and reports on flood disaster risk of the study area were used to address research objective 1. To address research objective 2, the DEM and country shapefile of Ghana were used to analyse the spatial distribution of flood emergency evacuation sites for flood-prone neighbourhoods in the Accra Metropolitan Assembly. For standardisation of the data set, all the data gathered were converted into raster data for the analysis. This was followed by reclassification of the layers, weighting them and then overlaying the output layers with background information such as the DEM to see the best potential sites. The study further applied the weighted linear combination (WLC) method to estimate site suitability for emergency evacuation sites. Weighted linear combination is a multicriteria evaluation and ranking method used in GIS-based studies such as site suitability, site selection and resource evaluation analysis (Ayalew, Yamagishi & Ugawa 2004; Basnet, Apan & Raine 2001; Malczewski 2000). The method can use both a quantitative approach and a qualitative approach. Once the factors were determined, a rule-based weight-selection technique dependent on selected criteria was used to determine the ratings for values of the factors. In the qualitative phase, an opinion-based subjective technique was used to develop weights for each variable (Ayalew et al. 2004; Malczewski 2000). Finally, the factor ratings of each variable were multiplied with their respective weights, and all the weighted layers were combined through addition to estimate suitability scores for each land unit. The WLC method was selected because it is a comprehensive suitability analysis method that combines terrain and relevant factors for selection and estimation of suitable locations for siting of facilities:

$$\text{Score}=\left(\sum_{j}^{n}FR_{j}\times W_{j}\right)$$ [Eqn 1]

Where Score = summary suitability score for the location, *FR_j_ = Factor Rating for factor j, n* = number of factors included in the model and *Wj* = weight assigned to factor *j* such that:

$$\sum_{j}^{n}Wj\times 100.$$ [Eqn 2]

### Data and variables

Siting of evacuation centres depends on both social and physical factors. However, some factors are necessary and critical for the siting of an evacuation centre. Specification of the factors usually depends on the type of disaster in question. For instance, specific parameters are required for the siting of an evacuation centre in order to deal with the situation during a particular type of disaster. Disasters such as earthquake, fire, hurricanes and flooding require emergency evacuation sites that need to be strategically located. For this reason, highland areas are considered a critical potential parameter in the event of an emergency situation because of flooding. For this study, the terrain of the study area was considered a critical factor for the siting of emergency evacuation centres that will cater for emergency situations during flooding. Both physical and social factors influence the site suitability of an evacuation centre. However, based on the type of disaster at the study site, a research which has analysed and established the criteria for flood emergency evacuation sites is unclear.

The land use types, road network, proximity of the site to flood zones and DEM of the study area were considered useful for analysis. Following the WLC method for site selection, the factors for suitability analysis were given weights and ranked in order of importance of each of the factors. The weights assigned to these factors were elevation 50%, land use 20%, road network 15% and flood-prone areas 15%. Elevation was considered most important to have at 50% because it is a baseline for establishing safe havens during flooding situations. Next in the order of importance is land use, which was ranked 20%. Land use is given this rank because different land uses offer various ecological functions and importance. Thus, not every land use can be destroyed in order to site an evacuation centre. Road network and flood-prone areas were given 15% each, indicating similar ranks for importance. Road networks play important functions for evacuating life and property to safe havens, and it is critical to consider nearness to roads in the siting of emergency centres. Flood-prone areas signify settlements that are vulnerable to flood hazards. Thus, emergency evacuation centre sites need to be located near flood-prone areas of the study site.

### Data processing

**FIGURE 2:**
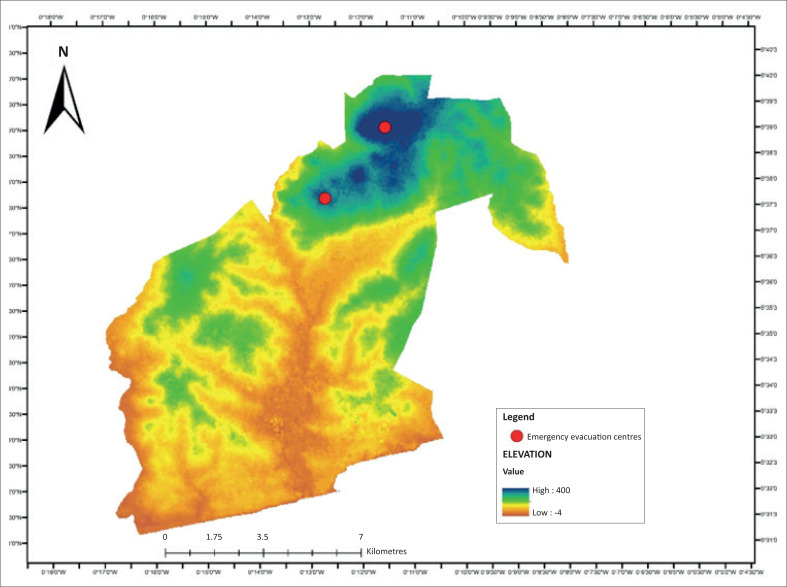
Evacuation sites.

Four data sets were used in this study to locate suitable areas for emergency evacuation sites during flood disasters as the main objective of the research. The majority of the study area is in flood-prone zones, so the driving factor for the siting of an evacuation centre in such an environment is the terrain of the study area. Other driving factors that were considered include the flood-prone areas within the study area, the land use types and road network (Table 1). Thus, the study used four criteria, which consist of land use, elevation, flood-prone areas and road network. These data were derived from the base data (e.g. the Euclidean distance to flood areas within the study area was determined from the flood-prone area base data for the submodel). From the derived data step, the data were then transformed and weighted. Next, the model was then run to locate the suitable sites for emergency evacuation sites (Figure 2).

**TABLE 1:** The geospatial data used in this study.

Data	Description	Format	Source
Land use	Urban areas, crop land, forest, water bodies	Raster	Interpretation of Landsat satellite data by authors
Elevation	Digital elevation model	Raster	United States Geological Survey (USGS) Earth Explorer website
Flood-prone areas	Refers to high risk areas of flooding within the study area	Vector shapefile	Nyarko 2000, 2002
Roads	Road network of the study area	Vector shapefile	Centre of remote sensing and geographical information systems (CERGIS), University of Ghana

### Ethical considerations

This study was conducted based on review on the rigorous literature review and GIS data. All academic research protocols including all works and data used in this article were acknowledged and cited. No plagiarism was performed and the article is mainly for academic purposes. This article did not involve animals and human subjects or participants and therefore did not need ethical clearance.

## Results and discussion

### Flood risk zones of the study area

**FIGURE 3:**
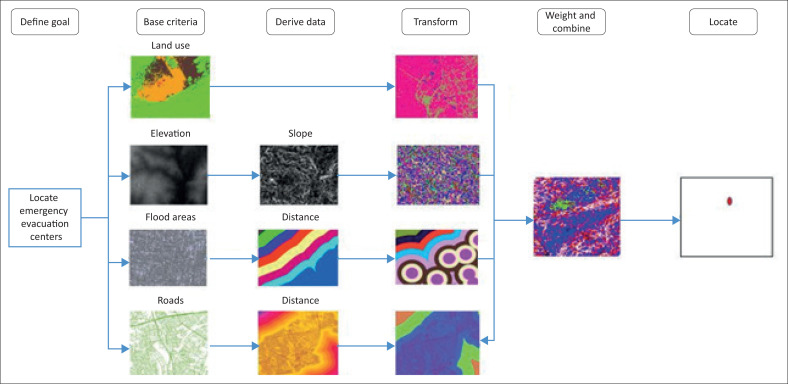
Workflow of data processing.

**FIGURE 4:**
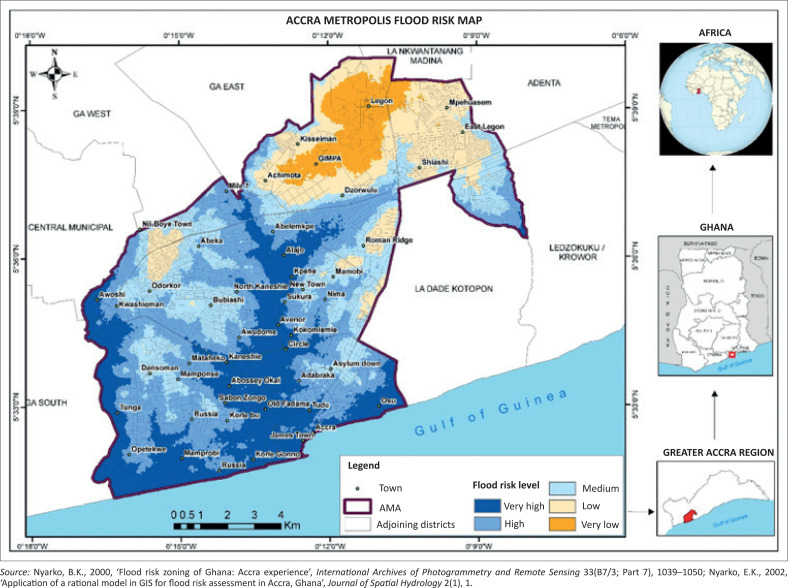
Flood-risk zones of the study area.

Flood risk describes an overlapping area of flood hazard and flood vulnerability (Nyarko 2000, 2002). Figure 3 shows five regions of very high, high, medium, low and very low risk of flood zones. Figure 3 indicates that the whole of the AMA is a flood-risk zone, and the level of risk to flooding varies. The very high flood risk zone (coloured red) covers Ashale-Botwe, Ashaiman, Frafraha and the adjoining neighbourhoods. These areas fall within high flood-risk zones, marked with yellow, and the medium risk zones fall in the eastern corner of the AMA, as indicated in Figure 4.

### Identification of evacuation sites

**FIGURE 5:**
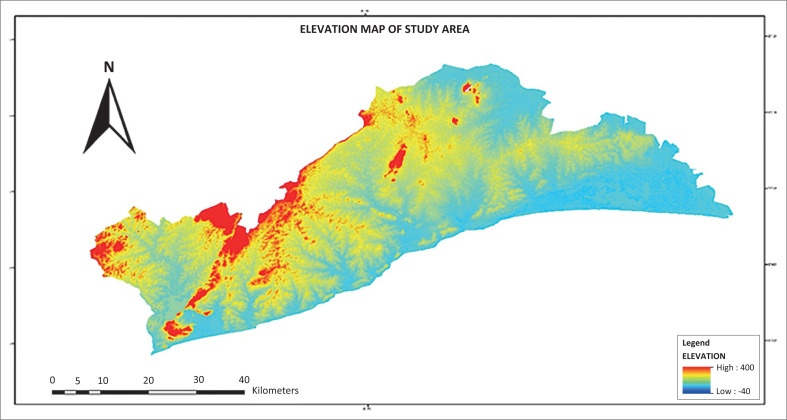
Digital elevation map of the study area.

A digital elevation model is useful for topographical analysis and important for identification of lowlands and highlands. This is an initial step in gauging where evacuation sites can be located in the study area. Emergency evacuation sites are usually on high ground, safe from flood hazards, and this is a criterion for selecting high elevations for this research. Figure 5 shows a digital elevation map of the study area, with the lowest point being −40 m below sea level and the highest point being 400 m above sea level. The distribution of the areas with potential as safe havens within the 400 m are not evenly distributed on the map, as they are concentrated along the north-western, northern and central parts of the map. The yellow label shows highland areas are between 300 m and 360 m on the map. These areas are concentrated along the northern part of the map, showing the potential to have elevation points near the flood-prone areas identified. This area has the elevation for potential siting of evacuation sites, because it falls outside flood-prone elevations.

In determining the locations for the evacuation sites, a set of criteria for site selection and suitability analysis was considered in this research. Several factors such as high elevation, flood-prone communities and access to transportation networks, health facilities and other facilities were given weights in selecting sites for emergency evacuation sites (Mustaffa et al. 2015). In this research, the most important factors for demarcating areas for flood emergency centres were high elevation (between 300 m and 400 m above sea level) with very low flood risk and adjacency of such highland areas to flood-prone communities in the AMA.

On this basis, one can argue from Figure 4 that the potential for flood emergency evacuation sites lies between 300 m and 400 m above sea level. Potential evacuation sites therefore can fall within the contours of 300 m and 400 m, covering the yellow and red regions of Figure 5.

### Flood-prone settlements and closest emergency evacuation sites

Emergency evacuation sites are part of disaster-risk management planning, and this project focuses on mapping of potential sites for evacuation of property and victims of flood disasters. The results revealed that flood occurs in lowland areas of settlements within drainage basins of the study site. Figure 5 shows the settlements, elevation, drainage (stream networks) and potential evacuation sites across the GAR. Settlements are labelled with dots, and the flood-prone communities of the settlements are located adjacent to the main streams. The highest elevation of 400 m above sea level shows the upper limit, and the 300 m of elevation marked in yellow shows the lower limit for consideration in the demarcation of emergency sites. Emergency evacuation sites need to consider proximity to flood disaster-prone areas to ensure quick evacuation of people and property during flood events. Areas in higher elevations than usually flood-prone communities have potential to be considered for such sites. Flood predominantly occurs along areas below the 300-m contour; thus, it makes sense to site flood emergency evacuation sites between 300 m and 400 m.

**FIGURE 6:**
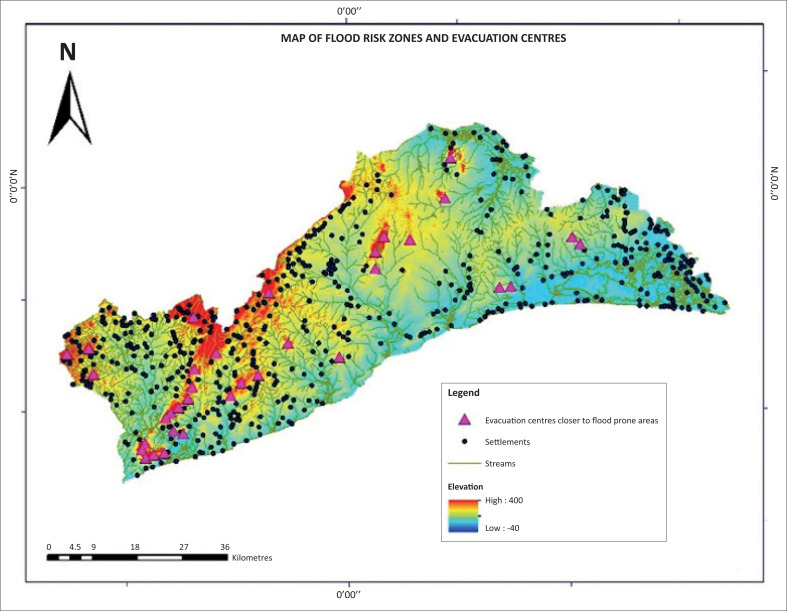
Flood-risk zones and closest evacuation sites for flood-prone communities.

The flood-prone communities are along the stream networks. The south-western part of Figure 6 is the AMA (see Figure 2) and it is generally a low-lying area, with settlements located at the dotted symbols along the stream networks and areas prone to floods. The 300-m contour serves as the baseline for determining emergency evacuation sites near flood-prone settlements. This is because all the other factors such as land use and road network are important, but high elevation and proximity to flood risk zones are crucial in determining emergency evacuation sites during flooding. Figure 6 shows the high points of 400 m, indicated with triangular symbols, as evacuation sites and their surrounding areas within elevation of 300 m above sea levels. These places are higher grounds, closest to areas that experience flooding in the AMA. These sites fall outside areas marked low and very low flood risk zones, as indicated in Figure 4.

### Optimal evacuation site selection

**FIGURE 7:**
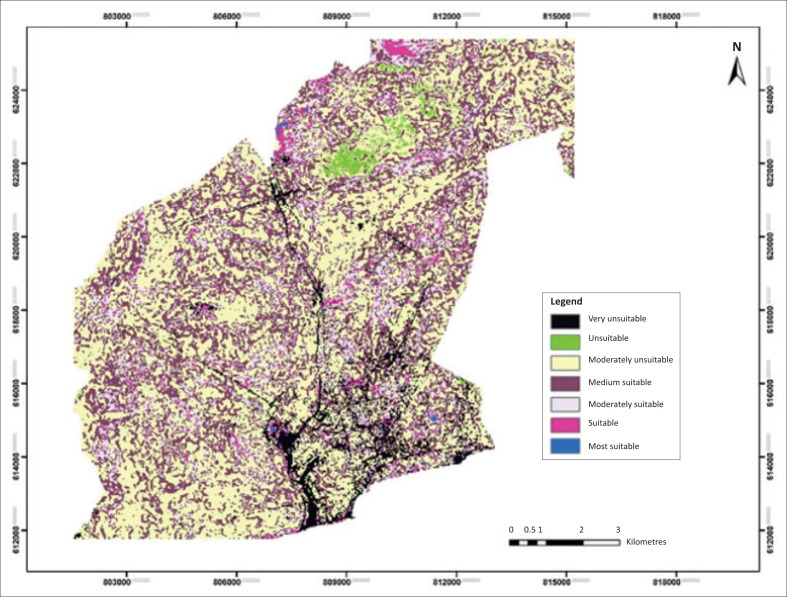
Suitability of sites for flood emergency evacuation centres in Accra in the metropolitan assembly.

In order to get the best sites for the evacuation centres, the DEM was considered together with the land use, flood-prone areas and road networks of the study area. Figure 7 shows the land suitability map for selecting the best possible sites for siting emergency evacuation centres within the study area in order to deal with the flood disaster situation. The land suitability map was divided into seven classes. These classes are the most suitable, suitable, moderately suitable, medium suitable, moderately unsuitable, unsuitable and very unsuitable sites. This classification was based on the cumulative evaluation of the criteria and weights given to the classes at a criterion level and the attributes considered in the classification.

In Figure 2, the very unsuitable sites are concentrated along the Odaw drainage basin and lagoons in the centre towards the southern part of the map. These areas constitute the core drainage network through which storm waters enter the sea. This is clearly linked to the distribution of lowland areas in Figure 5 and lack of settlements in these particular areas indicated in Figure 6. Again, the potential locations of emergency evacuation centres in Figure 2 also show no signs for siting of such centres. These areas are lowland areas below the 300-m contour and cannot be fit for locating the evacuation centres. The green portions in Figure 7 are concentrated in the north-eastern part and scattered along the south-western parts of the figure showing the unsuitable locations, as these areas are prone to flood risk and also fall below the 300-m contour. The moderately unsuitable regions are along these areas and may have settlements prone to floods, but elevation factors and nearness to transportation networks do not fit the criteria. The medium suitable zone also lacks the factors for the suitability analysis criteria. The moderately suitable sites have most of the factors, but the high elevation (which is critical for siting of a safe haven) falls below the 300-m contour, which is not safe for extreme flood risk. The suitable and most suitable zones are considered the best areas which fall within the 300 m and 400 m contour zones that have infrastructural facilities and settlements that face flood risk. The presence of these factors meets the suitability analysis and site selection standards for locations suitable for siting emergency evacuation centres in the AMA. Similar studies acknowledge the importance of emergency evacuation sites for emergency flood disaster response. Mustaffa et al. (2015) applied GIS and remote sensing to conduct a similar study in Malaysia and concluded that factors such as location, elevation and flood-prone areas are important for mapping flood emergency evacuation sites. In addition, Kourgialas and Karatzas (2011) gave importance to highland areas above the elevation of flood risk zones in selecting emergency evacuation sites for emergency flood response planning. Studies asserted the importance of GIS modelling for flood risk management and emergency response planning (Jamrussri & Toda 2018). Geographic information systems are useful for spatial analysis of flood hazards, vulnerability and disaster risk management, and studies have employed this approach for similar studies (Chang 2019).

## Conclusion

This research used GIS to map flood emergency evacuation sites of flood-prone neighbourhoods in the AMA in the GAR. Flood-prone communities of the study area were identified following the settlement map and confirmation of studies that mapped out the flood risk zones of the AMA. The DEM of the study site was then developed to show the topography and the stream networks of the study site. The highland areas within and around the flood-prone neighbourhoods were identified as points for emergency evacuation during flood events in the AMA. The distribution of these was interpreted to address the objectives of this research.

The results of this research are relevant for emergency flood risk management planning and allocation of emergency evacuation sites for flood-prone areas of the AMA. The research can also provide relevant literature for future research on GIS analysis of flood risk management of the study site and other areas with similar issues. A key finding of this study is that emergency evacuation sites are not evenly distributed across the study site. The potential points for location of evacuation sites are in the eastern, northern and western parts of the AMA.

### Limitations

The researchers acknowledge that this study has limitations that need to be outlined. The research was limited to flood-prone neighbourhoods of the AMA in the final analysis and only employed GIS tools for the study. The study relied on secondary data from existing sources. Although the secondary data came from reliably credible sources, primary data would have complimented the secondary data for a more comprehensive analysis.

### Recommendations

From the results and the limitations of this study, the following recommendations can be made for emergency flood risk management planning policy and future research. For practical responses to emergency flood disasters in the AMA, this research recommends that emergency evacuation sites need to be established in and around elevations of 300 m – 400 m above sea level as safe havens. These elevation points fall within very low flood risk zones in the AMA. It is recommended that future research should consider primary and secondary data for a more comprehensive research on the topic. Pragmatic and more rigorous field work can be combined with the GIS modelling for more comprehensive and relevant flood risk decision-making purposes.
